# Knockdown of growth factor receptor bound protein 7 suppresses angiogenesis by inhibiting the secretion of vascular endothelial growth factor A in ovarian cancer cells

**DOI:** 10.1080/21655979.2021.2005225

**Published:** 2021-12-07

**Authors:** Qiong Xu, Zequn Liu, Zhi-qin Zhu, Yue Fan, Rui Chen, Xiao-hui Xie, Mi Cheng

**Affiliations:** a Department of Gynaecology, Guangzhou Women and Children’s Medical Center, Guangzhou Medical University, Guangzhou, Guangdong, China; b Department of Prenatal Diagnostic Center, Guangzhou Women and Children’s Medical Center, Guangzhou Medical University, Guangzhou, Guangdong, China; c Department of Obstetrics, Guangzhou Women and Children’s Medical Center, Guangzhou Medical University, Guangzhou, Guangdong, China

**Keywords:** Ovarian cancer, GRB7, angiogenesis, co-culture, HUVECS

## Abstract

Growth factor receptor bound protein 7 (GRB7) plays an important role in regulating the growth and metastasis of ovarian cancer. Angiogenesis is the basis for the growth, invasion, and metastasis of malignant tumors. In the current study, we aimed to determine whether GRB7 plays a role in regulating angiogenesis in ovarian cancer. Immunohistochemistry on tissue microarray showed that GRB7 and platelet endothelial cell adhesion molecule-1 (PECAM-1/CD31) protein expression were positively correlated in ovarian cancer tissues. *GRB7* knockdown suppressed vascular endothelial growth factor A (VEGFA) expression and reduced VEGFA secretion. The effects of *GRB7*-silenced SKOV-3 cells on human umbilical vein endothelial cells (HUVECs) were evaluated using a transwell cell co-culture model, which showed that knockdown of *GRB7* in SKOV-3 cells suppressed HUVEC proliferation, migration, invasion, and tube formation. Moreover, knockdown of *GRB7* in SKOV-3 cells downregulated the expression of proteins associated with angiogenesis, including vascular endothelial growth factor receptor-2 (VEGFR2), mitogen-activated protein kinase kinase 1 (MAP2K1/MEK1), extracellular signal-regulated kinases 1 and 2 (ERK1/2), notch receptor 1 (NOTCH1), and delta-like canonical Notch ligand 4 (DLL4) in HUVECs. In conclusion, knockdown of *GRB7* in ovarian cancer cells is an attractive potential therapeutic target for the suppression of angiogenesis in ovarian cancer. GRB7 may regulate angiogenesis through VEGFA/VEGFR2 signaling and its downstream pathways.

## Introduction

According to the International Agency for Research on Cancer in 2020 (https://gco.iarc.fr/today/fact-sheets-cancers), ovarian cancer is the third most common gynecological malignancy. Its age-standardized incidence rate was 6.6 per 100,000 worldwide, and its age-standardized mortality rate was 4.2 per 100,000 worldwide. The 5-year survival rates of patients with stage III and IV ovarian cancer are 41% and 20%, respectively [1]. Although >80% of patients with stage III and IV ovarian cancer respond to surgery and adjuvant chemotherapy, approximately 80% of these patients experience a relapse within 18 months [2–4]. Therefore, there is an urgent need to develop new, effective treatment options to curb its recurrence and metastasis, improve the survival status of patients with advanced disease, and increase survival.

With advancements in research on tumor molecular biology, targeted therapy is expected to be a more effective and less toxic treatment strategy for patients with ovarian cancer [5,6]. Angiogenesis is an important aspect affecting both tumor growth and metastasis; its extent directly affects the clinical outcome of cancers [7–9]. Several studies have shown that angiogenesis promotes the development, progression, and metastasis of ovarian cancer [8,10]. Anti-angiogenic therapy is a major research focus for tumor-targeted therapy [11]. At present, some anti-angiogenesis targeted drugs, such as bevacizumab, have been used to treat ovarian cancer and have improved patient outcomes [12]. Nonetheless, resistance to anti-angiogenesis-targeted drugs is inevitable. Therefore, further research on ovarian cancer angiogenesis is indispensable to discover new targets for the development of new targeted therapeutics.

Growth factor receptor bound protein 7 (GRB7) is an important adaptor protein that is primarily involved in regulating tumor invasion and metastasis, as well as mammalian embryo migration [13]. Multiple studies have found that GRB7 is highly expressed in various tumor tissues, including ovarian cancer tissues [13–16]. *In vitro* experiments have shown that silencing the expression of GRB7 can significantly inhibit the migration and invasion of ovarian cancer cells [17]. A study using clinical samples suggested that the GRB7/extracellular signal-regulated kinases (ERK)/forkhead box M1 (FOXM1) signaling cascade is a promising molecular therapeutic target for ovarian cancer [18]. These data suggest that GRB7 plays an important role in regulating the occurrence and development of ovarian cancer. Although these studies have reported the function of GRB7, the mechanism of action is not yet fully understood, with more in-depth research still needed.

In view of the important role of angiogenesis in ovarian cancer growth and metastasis, we hypothesized that GRB7 might promote the growth and metastasis of ovarian cancer cells by regulating angiogenesis to increase their blood supply. In the present study, we have assessed the role of GRB7 in angiogenesis using clinical samples and an experimental cell-culture model, implicating the angiogenesis marker protein, platelet endothelial cell adhesion molecule-1 (CD31) [19].

## Materials and methods

### Chemicals and reagents

Sodium chloride, anhydrous ethanol, ethanol, methanol, anhydrous sodium sulfite, sodium thiosulfate, and glacial acetic acid were purchased from Guangzhou Chemical Reagent (China). Diethyl Phosphate buffer solution (PBS, pH 7.4), pyr-ocarbonate (DEPC) and polyoxyethy-lene sorbitan monolaurate (Tween-20) were purchased from Sangon Biotech (Shanghai, China). Other chemicals and reagents were all from Sigma (St. Louis, MO, USA).

### Measurement of GRB7 and CD31 protein levels

The ovarian cancer tissue microarray was purchased from the Shanghai Outdo Biotech Company (Shanghai, China). The serial number was HOvaC070PT01. This microarray included 65 ovarian cancer tissue samples. Immunohistochemistry to detect GRB7 and CD31 was performed by the Shanghai Outdo Biotech Company using conventional protocols [20]. The dilution of anti-GRB7 antibody (catalog number: 10,045-1-Ig, Proteintech Group, Inc., Rosemont, IL, USA) was 1:200. The dilution of anti-CD31 antibody (Catalog number: 11,265-1-AP, Proteintech Group, Inc.) was 1:1000. After excluding samples that detached from the glass and those with few tumor cells, 60 ovarian cancer tissues met the requirements of analysis.

### Correlation between GRB7 and CD31 expression

GRB7 and CD31 protein expression levels were scored according to the method described by Bollag et al. [21]. The degree of staining was quantified by scoring for intensity (non-stained, 0; weakly stained, 1; moderately stained, 2; strongly stained, 3) and extent of staining (0–100%). The percentage of positively stained tumor cells was multiplied by the degree of staining. The staining score was calculated by multiplying the staining intensity score by the staining extent score (out of a maximum of 300%). The correlation between the expression levels of GRB7 and CD31 was analyzed using the linear regression analysis tool of GraphPad Prism version 7.0 (GraphPad Software, San Diego, CA, USA).

### Cell culture

The human ovarian cancer cell line SKOV-3 cells were cultured in McCoy’s 5A medium (Gibco, Logan, UT, USA) containing 10% fetal bovine serum (Gibco). Human umbilical vein endothelial cells (HUVECs) were cultured in Ham’s F12 K medium (Gibco) with 10% fetal bovine serum, 100 μg/ml heparin (Gibco), and 50 μg/ml endothelial cell growth supplement (Sigma, St. Louis, MO, USA). All cells were cultured at 37 °C in a humidified atmosphere containing 5% CO_2_. All cells were purchased from the Cell Bank of the Chinese Academy of Sciences (Shanghai, China).

### Transfection of small interfering ribonucleic acids (siRNAs)

Two siRNAs (siGRB7-1 and siGRB7-2) targeting two sites of the *GRB7* coding region (siGRB7-1 targeting ccttgagaagtgcctcagata and siGRB7-2 targeting cgccaagtacgaactgttcaa) were designed and synthesized by GenePharma Co., Ltd. (Suzhou, China). Negative control siRNA (NC) was used as a control. NC, siGRB7-1, and siGRB7-2 was transfected into SKOV-3 cells using Lipofectamine RNAiMAX according to the manufacturer’s instructions (Invitrogen, Carlsbad, CA, USA). Briefly, SKOV-3 cells were seeded in 6-well plates. RNA-lipid complexes were prepared according to the manufacturer’s instructions. When cell confluence reached approximately 70%, 250 μl RNA-lipid complexes containing 25 pmol siRNA and 7.5 μl Lipofectamine RNAiMAX reagent were added to the cells. After culturing at 37 °C in a humidified atmosphere containing 5% CO_2_ for 24 or 48 h, cells were used for subsequent assays.

### Quantitative reverse transcription PCR (qRT-PCR)

At 24 h after transfection, SKOV-3 cells were harvested using a cell scraper for qRT-PCR analysis [22]. Total RNA was extracted using the TRIzol reagent (Invitrogen). EasyScript First-Strand cDNA Synthesis SuperMix was used for reverse transcription. The PCR mixture was prepared using SYBR Green qPCR SuperMix (Vazyme Biotech Co., Ltd., Nanjing, China). PCR was performed using an ABI PRISM® 7500 Sequence Detection System (Foster City, CA, USA). The primer sequences (5ʹ-3ʹ) for detecting *GRB7* were TGCAGTACGTGGCAGATGTG (Tm 60°C) and GAAGATCCGAAGCCCCTTGT (Tm 59°C). Primers for *GRB7* were designed using Primer Premier 5.0 (PREMIER Biosoft International, Palo Alto, CA, USA). The primer sequences (5ʹ-3ʹ) used as an internal expression control (18S ribosomal RNA) were CCTGGATACCGCAGCTAGGA and GCGGCGCAATACGAATGCCCC [23].

### Western blotting

At 48 h after transfection, SKOV-3 cells were harvested using a cell scraper for total protein isolation. Western blotting was performed as described by Guo et al. [22]. All primary antibodies were purchased from Proteintech Group, Inc., and their catalog numbers and dilutions were as follows: anti-GRB7 (Catalog number 10,045-1-Ig; 1:1000), anti-vascular endothelial growth factor A (VEGFA) (19,003-1-AP, 1:2000), anti-vascular endothelial growth factor receptor-2 (VEGFR2) (26,415-1-AP, 1:800), anti-mitogen-activated protein kinase kinase 1 (MAP2K1/MEK1) (28,930-1-AP, 1:3000), anti-extracellular signal-regulated kinases 1 and 2 (ERK1/2) (28,733-1-AP, 1:4000), anti-notch receptor 1 (NOTCH1) (20,687-1-AP, 1:1000), anti-delta-like canonical Notch ligand 4 (DLL4) (21,584-1-AP, 1:600), and anti-glyceraldehyde-3-phosphate dehydrogenase (GAPDH) (10,494-1-AP, 1:6000). GAPDH was used as the loading control.

### Enzyme-linked immunosorbent assay (ELISA)

Culture supernatants of SKOV-3 cells were collected in centrifuge tubes. After centrifugation at 1000 × g for 20 min to remove cell debris and impurities, the supernatant was sampled for ELISA to measure extracellular of VEGFA. ELISA was performed according to the manufacturer’s instructions of human VEGFA ELISA kit (ab119566, Abcam Company, Cambridge, MA, USA).

### Co-culture of SKOV-3 and HUVECs

SKOV-3 cells were transfected with siRNAs. At 24 h after transfection, they were harvested using 0.25% trypsin digestion for co-culture with HUVECs in a 24-well transwell system (Corning Incorporated, Corning, NY, USA) [24]. For the Cell Counting Kit-8 (CCK8) assay, diamidinyl phenyl indole (DAPI) staining, scratch wound healing assay, and Matrigel tube formation asasy, HUVECs were seeded in the lower chamber of the transwell system, and SKOV-3 cells transfected with either NC, siGRB7-1, or siGRB7-2 were seeded in the upper chamber of the transwell system. For transwell migration and invasion assays, HUVECs were seeded in the upper chamber, and SKOV-3 cells transfected with either NC, siGRB7-1, or siGRB7-2 were seeded in the lower chamber. HUVECs co-cultured with SKOV-3 cells transfected with different siRNAs were named HUVECs-NC, HUVECs-siGRB7-1, and HUVECs-siGRB7-2.

### CCK8 assay

Co-culture was performed as described previously. The assay was performed according to the manufacturer’s instructions using a CCK8 kit (Jiangsu KeyGEN BioTECH, China) [25]. After co-culture for 0, 1, 2, or 3 days, 100 μl CCK8 solution (10% volume of culture medium) was added to the lower chamber of the transwell system. After incubation at 37 ℃ in a humidified atmosphere containing 5% CO_2_., optical density (OD) was measured at 450 nm using a microplate reader (Multiscan MK3; Thermo Fisher Scientific, Waltham, MA, USA). The proliferation rate was calculated using the following formula:

$$\left(\frac{OD\;value\;of\;test\;group}{OD\;value\;of\;NC\;group\;at\;day\;0}\;-1\right)\times 100$$

### DAPI staining

After 48 h of co-culture, DAPI staining was carried out as described by Chazotte [26]. HUVECs-NC, HUVECs-siGRB7-1, or HUVECs-siGRB7-2 on glass slides were washed three times with phosphate-buffered saline (PBS). Cells were fixed with 3.7% formaldehyde for 10 min and rinsed three times with PBS. Next, the cells were treated with 0.2% Triton X-100 for 5 min to permeabilize the cells. After rinsing three times with PBS, cells were incubated with DAPI staining solution (Boster, Wuhan, China) for 10 min. After washing three times with PBS, the cells were covered with antifade mounting medium (Beyotime, Shanghai, China) and observed using a fluorescence microscope.

### Scratch wound healing assay

After 24 h of co-culture, the assay was performed following the method reported by Dai et al. [27]. The wound healing rate was calculated using the formula: (1-wound area at _each time point_ *÷* wound area _0 h_) *×* 100% (same sample).

### Migration and invasion assays

Co-culture of HUVECs and SKOV-3 cells was performed as described above. Transwell migration and transwell Matrigel invasion assays were performed as described by Guo et al. [22]. The upper chamber was removed, and the medium was aspirated after 24 h of co-culture. Cells on the inner surface of the upper chamber that did not cross the polycarbonate membrane were gently wiped with a cotton swab. Next, the upper chamber was placed in a new 24-well plate with 600 μL of 4% paraformaldehyde and fixed for 20–30 min. After discarding the fixative, cells were stained with 0.1%-0.2% crystal violet for 10 min and washed three times with phosphate-buffered saline to remove unbound crystal violet. The dye on the inner surface of the upper chamber was wiped with a cotton swab. After air-drying, eight fields of view were randomly selected and analyzed under a light microscope. The average number of counted cells was used to assess the migration and invasion capabilities. For the transwell invasion assay, the inner surface of the upper chamber was coated with Matrigel (BD Biosciences), and the migration assay was performed using the same protocol as described for the migration assay.

### Matrigel tube formation assay

The Matrigel tube formation assay was performed as described by Shi et al. [25]. after 24 h of co-culture at 37°C in a 5% CO_2_ incubator, tube formation in HUVECs was observed under a microscope (40x) and photographed. The number of tubes in each image was counted, and the average number was used to evaluate HUVEC tube formation.

### Statistical analysis

The results from three independent experiments are shown as means ± standard deviation. Differences between two groups were analyzed using *t*-tests performed using GraphPad Prism (version 7.0, GraphPad Software, San Diego, CA, USA). Correlations between expression levels of CD31 and GRB7 in ovarian cancer tissues was analyzed by linear regression analysis using GraphPad Prism version 7.0. Statistical significance was set at *P* < 0.05.

## Results

We hypothesized that GRB7 may play an important role in angiogenesis in ovarian cancer. To test this hypothesis, we first analyzed the expression of GRB7 and CD31 proteins in ovarian cancer tissues by immunohistochemistry. Furthermore, we analyzed the effect of *GRB7* knockdown in SKOV-3 cells on the proliferation, migration, invasion, and tube formation of HUVECs, as well as on the expression levels of proteins associated with angiogenesis in HUVECs using a transwell co-culture model.

### The protein expression of GRB7 and CD31 was positively correlated in ovarian cancer tissues

**Figure 1.**
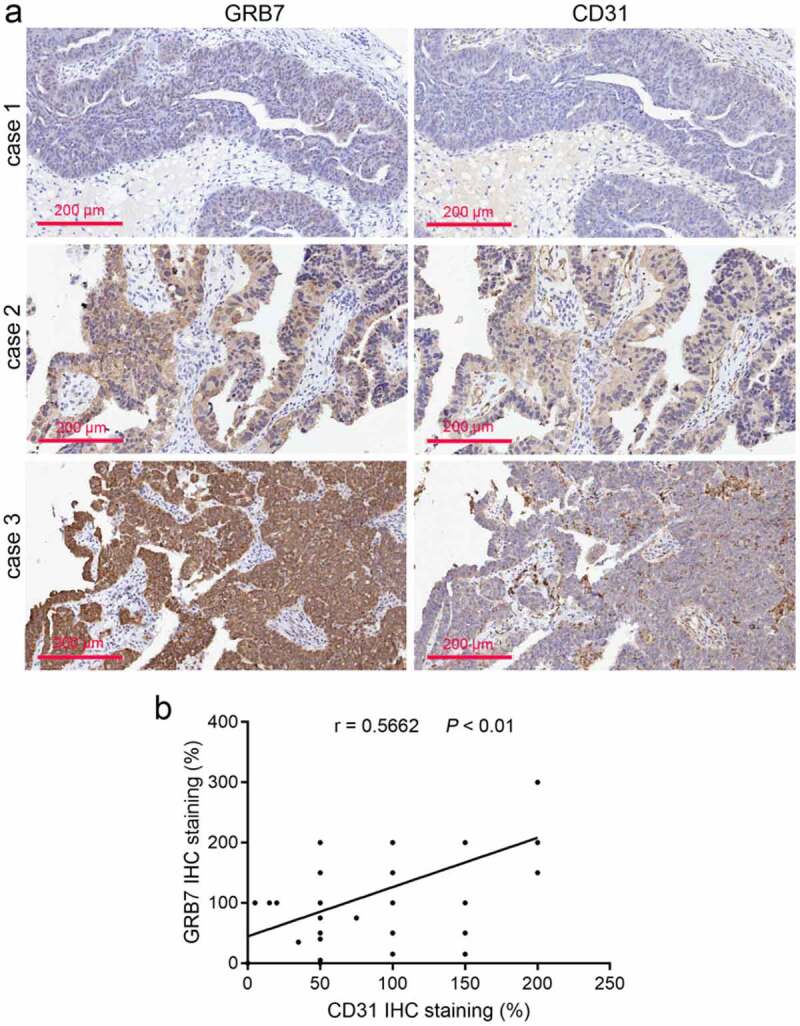
GRB7 and CD31 expression is positively correlated in ovarian cancer tissues (a) Immunohistochemical images of GRB7 and CD31 in ovarian cancer tissues from three different patients. Images are magnified at 200x. b: The positive correlation between GRB7 and CD31 expression (*n* = 60 per group) was analyzed using linear regression analysis. r, correlation index

The protein expression of GRB7 and CD31 in ovarian cancer tissues was analyzed using immunohistochemistry. By imaging the same site in the same cancer tissue, we found that the expression of GRB7 and CD31 was significantly correlated (Figure 1a). To analyze their correlation more accurately, their expressions were scored using linear regression analysis. As shown in Figure 1b, the protein expression of GRB7 and CD31 was positively correlated (r = 0.5662, *P* < 0.01). Despite this observation, we found that the regions highly expressing GRB7 and CD31 in ovarian cancer tissues were different (Figure 1a).

### *Knockdown of* GRB7 *decreases intracellular and extracellular levels of VEGFA in SKOV-3 cells*

**Figure 2.**
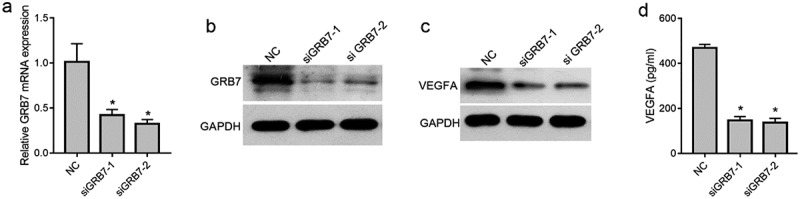
Knockdown of *GRB7* decreases intracellular and extracellular levels of VEGFA in of SKOV-3 cells. (a-b) *GRB7* was silenced using two siRNAs targeting *GRB7* (siGRB7-1 or siGRB7-2). NC, transfected with negative control siRNA. a: *GRB7* mRNA expression measured by qRT-PCR. b: GRB7 protein expression measured by western blotting. c: Knockdown of *GRB7* suppression of expression of intracellular VEGFA measured by western blotting. d: Knockdown of *GRB7* reduces the secretion of VEGFA as measued by ELISA. Bar graphs show means ± standard deviations from three independent experiments. *P < 0.05 compared to NC

To avoid off-target effects, two *GRB7* siRNAs targeting different sequences of the GRB7 coding region were transfected into SKOV-3 cells to knock down *GRB7* expression. As shown in Figures 2a and 2b, both mRNA and protein levels of GRB7 were significantly decreased by the two siRNAs. In addition, the intracellular and extracellular levels of VEGFA in SKOV-3 cells were assessed by western blotting and ELISA. Compared to the NC group, the levels of intracellular and extracellular VEGFA decreased distinctly in SKOV-3 cells transfected with siGRB7-1 or siGRB7-2 (Figures 2c and 2d), suggesting that knockdown of GRB7 not only suppressed the expression of VEGF, but also reduced the secretion of VEGFA.

### *Knockdown of* GRB7 *in SKOV-3 cells suppresses HUVEC proliferation*

**Figure 3.**
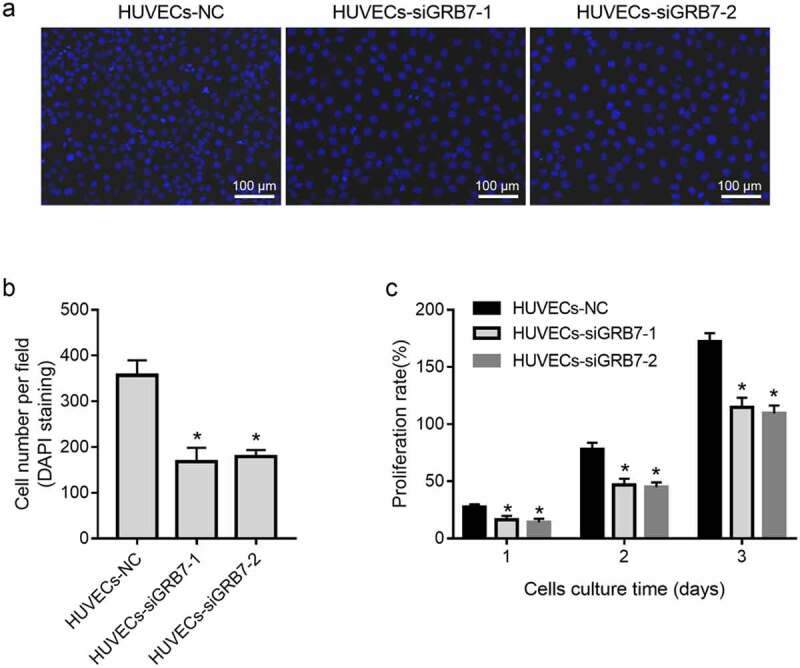
Knockdown of *GRB7* in SKOV-3 cells suppresses HUVEC proliferation. HUVECs were seeded in the lower chamber of a transwell insert and SKOV-3 cells transfected with negative control siRNA (NC) or siRNAs targeting *GRB7* (siGRB7-1 or siGRB7-2) were seeded in the upper chamber of the transwell insert. HUVECs co-cultured with SKOV-3 cells transfected with different siRNAs are designated as HUVECs-NC, HUVECs-siGRB7-1, and HUVECs-siGRB7-2. (a-b) DAPI staining showing that *GRB7* knokdown in SKOV-3 cells decreased the cell number per field. Images in panel a are magnified at 200x. (c) CCK8 assay showing that *GRB7* knokdown in SKOV-3 cells suppressed the proliferation of HUVECs. Bar graphs show means ± standard deviations from three independent experiments. *P < 0.05 compared to HUVECs-NC

Because of the dissimilar location of GRB7 and CD31 expression in ovarian cancer tissues, a contactless transwell co-culture system was designed to investigate the role of GRB7 in angiogenesis. First, we evaluated the effect of *GRB7* knockdown in SKOV-3 cells on HUVEC proliferation by DAPI staining and CCK8 assays. Cell numbers per field (Figures 3a and 3b) and the proliferation rate (Figure 3c) were reduced in both HUVECs-siGRB7-1 and HUVECs-siGRB7-2 cells than in the HUVECs-NC group, indicating that knockdown of GRB7 in SKOV-3 cells suppressed the proliferation of HUVECs.

### *Knockdown of* GRB7 *in SKOV-3 cells suppresses HUVEC migration and invasion*

**Figure 4.**
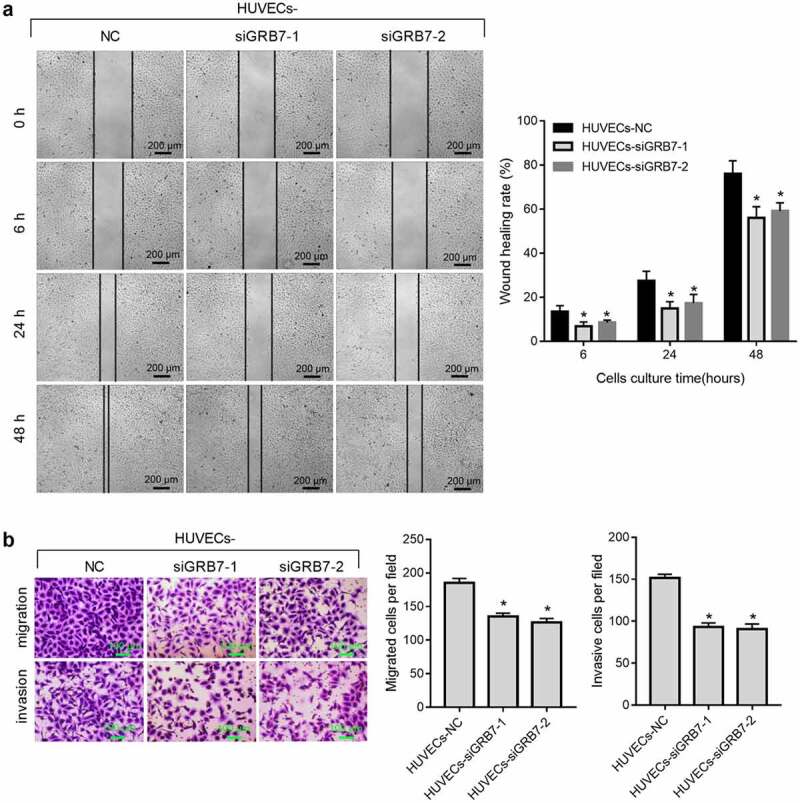
Knockdown of *GRB7* in SKOV-3 cells suppresses HUVEC migration and invasion. For the wound-healing assay, HUVECs were seeded in the lower chamber of a transwell insert, and SKOV-3 cells transfected with negative control siRNA (NC) or siRNAs targeting *GRB7* (siGRB7-1 or siGRB7-2) were seeded in the upper chamber of the transwell insert. For transwell migration and transwell-matrigel invasion assays, HUVECs were seeded in the upper chamber, and SKOV-3 cells transfected with NC, siGRB7-1 or siGRB7-2 were seeded in the lower chamber. HUVECs co-cultured with SKOV-3 cells are designated as HUVECs-NC, HUVECs-siGRB7-1, and HUVECs-siGRB7-2. (a) Knockdown of *GRB7* in SKOV-3 cells slows healing, indicating that it supresses migration. Images in panel a are magnified at 40x. (b) Knockdown of *GRB7* in SKOV-3 cells reduced both migrating and invading cell numbers. Images in panel b are magnified at 100x. Bar graphs show means ± standard deviations from three independent experiments.*P < 0.05 compared to HUVECs-NC

We further evaluated the effect of *GRB7* knockdown on the migration and invasion of HUVECs using scratch wound healing, transwell migration, and transwell invasion assays. Wound healing from 6 h to 48 h after scratching was slower (Figure 4a), and migrated or invasive cell numbers were lower (Figure 4b) in both HUVECs-siGRB7-1 and HUVECs-siGRB7-2 groups than in the HUVECs-NC group, indicating that knockdown of *GRB7* in SKOV-3 cells suppressed the migration and invasion of HUVECs.

### *Knockdown of* GRB7 *in SKOV-3 cells suppresses tube formation by HUVECs*

**Figure 5.**
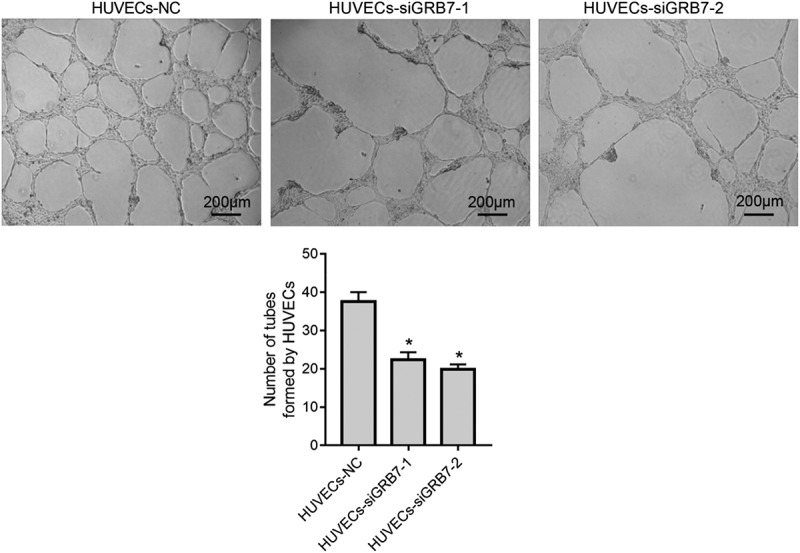
Knockdown of *GRB7* in SKOV-3 cells suppresses tube formation by HUVECs. HUVECs were seeded in the lower chamber of a transwell insert precoated with Matrigel, and SKOV-3 cells transfected with negative control siRNA (NC) or siRNAs targeting *GRB7* (siGRB7-1 or siGRB7-2) were seeded in the upper chamber of the transwell insert. HUVECs co-cultured with SKOV-3 cells transfected with different siRNAs are designated as HUVECs-NC, HUVECs-siGRB7-1, and HUVECs-siGRB7-2. After 6 h, fields were randomly selected and photographed. Images are magnified at 40x. Bar graphs show means ± standard deviations from three independent experiments. *P < 0.05, when compared to HUVECs-NC

In addition to proliferation and metastasis, we also evaluated the effect of *GRB7* knockdown in on tube formation using a Matrigel assay. The number of tubes formed by HUVECs was lower in both HUVECs-siGRB7-1 and HUVECs-siGRB7-2 groups than in the HUVECs-NC group (Figure 5).

### *Knockdown of* GRB7 *in SKOV-3 cells inactivates the expression of proteins associated with angiogenesis*

**Figure 6.**
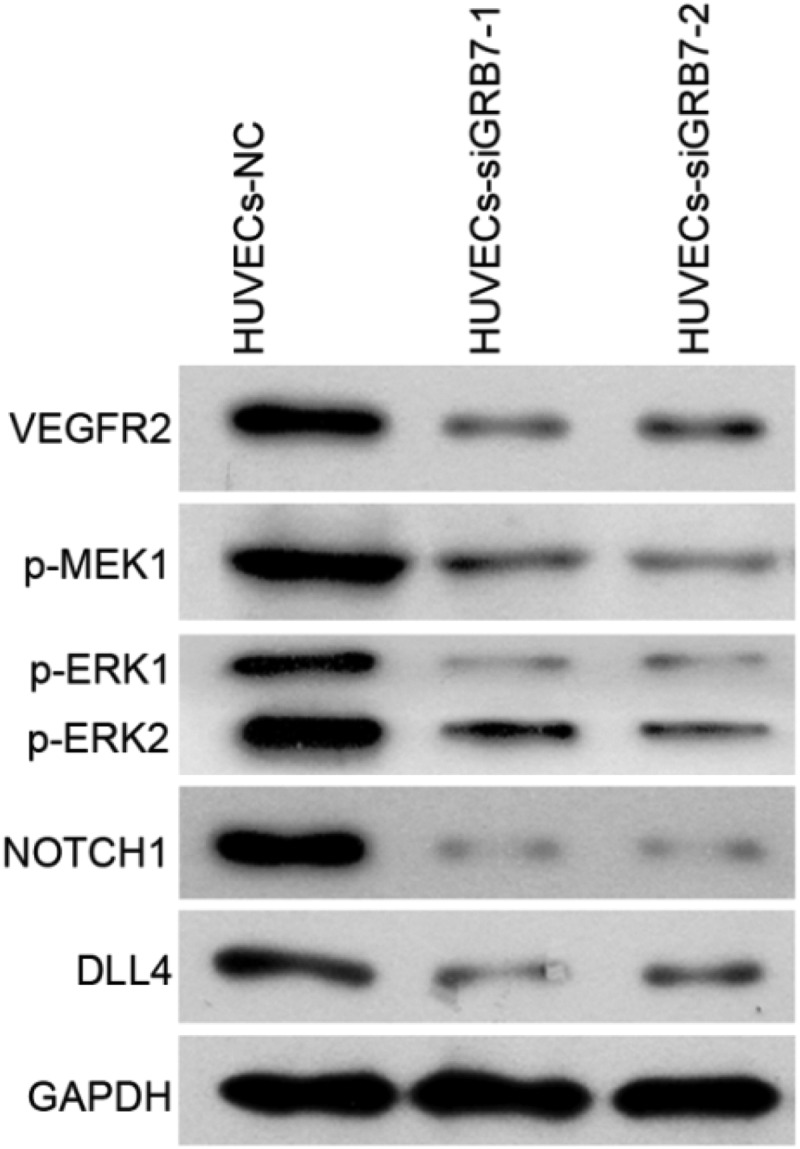
Knockdown of *GRB7* in SKOV-3 cells decreases the expression of proteins associated with angiogenesis. HUVECs were seeded in the lower chamber of a transwell insert, and SKOV-3 cells transfected with negative control siRNA (NC) or siRNAs targeting *GRB7* (siGRB7-1 or siGRB7-2) were seeded in the upper chamber of the transwell insert. These conditions are designated as HUVECs-NC, HUVECs-siGRB7-1, and HUVECs-siGRB7-2. After co-culture for 24 h, HUVECs were harvested for western blotting to measure the expression of VEGFR2, p-MEK1, p-ERK1/2, NOTCH1, and DLL4

To further characterize the mechanism of action, we analyzed the effect of *GRB7* knockdown on the angiogenesis-associated protein expression. As shown in Figure 6, the protein levels of VEGFR2, p-MEK1, p-ERK1/2, NOTCH1, and DLL4 were downregulated in both the HUVECs-siGRB7-1 and HUVECs-siGRB7-2 groups compared to those in the HUVECs-NC control cells (Figure 6).

## Discussion

To date, no study has reported a role for GRB7 in tumor angiogenesis. In this study, we observed an association between GRB7 and angiogenesis in ovarian cancer. Our data provide a new theoretical basis and research direction for the design and development of anti-vascular drugs for the treatment of ovarian cancer.

Both mRNA and protein levels of GRB7 are frequently upregulated in ovarian cancer samples compared to normal ovary control samples [16]. Platelet endothelial cell adhesion molecule-1 (CD31) is primarily expressed on the surface of endothelial cells, leukocytes, and platelets, and has been implicated in endothelial cell migration, angiogenesis, adhesion and accumulation of platelets, and inflammation [19,28]. CD31 is used as a marker to assess vessel density and tumor angiogenesis [29–31]. We found that GRB7 and CD31 expression levels in ovarian cancer tissues were significantly correlated. Therefore, we hypothesized that GRB7 may play a role in regulating angiogenesis in ovarian cancer.

Our hypothesis was further supported by data from a cell-culture model. First, we found that knockdown of GRB7 decreased the intracellular and extracellular levels of VEGFA in SKOV-3 cells. VEGFA is the most important vascular endothelial growth factor (VEGF) [32]. It has the highest specificity for inducing tumor angiogenesis [33,34]. Therefore, the suppressive effect of GRB7 on VEGFA is consistent with GRB7 playing a role in regulating angiogenesis in ovarian cancer. In addition, data from a non-contact transwell cell co-culture model showed that knockdown of *GRB7* in SKOV-3 cells suppressed HUVEC proliferation, migration, invasion, and tube formation. Tumor angiogenesis is a very complex process that generally includes vascular endothelial matrix degradation, endothelial cell migration, endothelial cell proliferation, vascular branching of endothelial cells to form vascular rings, and formation of new basement membranes [32,35]. Our results indicate that knockdown of *GRB7* in SKOV-3 cells suppresses endothelial cell function during angiogenesis. Moreover, *GRB7* knockdown downregulated the expression of proteins associated with angiogenesis, including VEGFR2, MAP2K1/MEK1, ERK1/2, NOTCH1, and DLL4. These findings further support our hypothesis that GRB7 plays a role in regulating angiogenesis in ovarian cancer. Currently, there are no reports regarding the role of GRB7 in regulating tumor angiogenesis. Our data suggest that GRB7 has potential as a target for the development of anti-angiogenic drugs.

We propose that GRB7 may regulate angiogenesis through VEGFA/VEGFR2 signaling. This hypothesis is supported by the following data: 1) the regions expressing high levels of GRB7 and CD31 in ovarian cancer tissues were different; 2) knockdown of *GRB7* decreased extracellular levels of VEGFA in SKOV-3 cells; 3) knockdown of *GRB7* in SKOV-3 cells decreased the protein levels of VEGFR2 in co-cultured HUVECs; and 4) Knockdown of *GRB7* in SKOV-3 cells decreased the protein levels of p-MEK1 and ERK1/2. Among the three high-affinity receptors of VEGFA, VEGFR-1/Flt-1, VEGFR-2/KDR/Flk-1, and VEGFR-3/Flt-4, VEGFR2 is the primary regulator of angiogenesis [33,36]. The MEK/ERK signaling pathway can be activated by VEGFA binding to VEGFR2 [36,37]. Therefore, GRB7 may function in angiogenesis through the VEGFA/VEGFR2/MEK/ERK signaling pathway.

Besides the VEGFA/VEGFR2/MEK/ERK signaling pathway, we also found that knockdown of *GRB7* in SKOV-3 cells decreased the expression of NOTCH1 and DLL4. DLL4 regulates the formation of tip cells during angiogenesis through Notch [38]. Both VEGF and Notch ligands are key players in angiogenesis that collaborate dynamically [39]. Moreover, other signals, such as JAK/STAT3 signaling, have roles in cancer angiogenesis [40]. The focus of this study was to analyze the role of GRB7 in angiogenesis, and the molecular mechanism is only preliminary. In future studies, we will further explore the mechanism of GRB7 action.

## Conclusion

In conclusion, GRB7 plays a role in regulating angiogenesis in ovarian cancer; reducing its expression may be a promising strategy to suppress angiogenesis in patients with ovarian cancer. GRB7 may play a role in angiogenesis through VEGFA/VEGFR2 signaling and its downstream pathways. In the present study, we did not explore the mechanism by which GRB7 regulates the expression and secretion of VEGFA in ovarian cancer cells. This is one of its limitations. We will address this issue in future studies.

## Data Availability

All data from this study are available in this published article.

## References

[1] Torre LA, Trabert B, DeSantis CE, et al. Ovarian cancer statistics, 2018. CA Cancer J Clin. 2018;68(4):284–296. 29809280 10.3322/caac.21456 PMC6621554

[2] Luvero D, Plotti F, Aloisia A, et al. Ovarian cancer relapse: from the latest scientific evidence to the best practice. Crit Rev Oncol Hematol. 2019;140:28–38. 31176270 10.1016/j.critrevonc.2019.05.014

[3] Lee JM, Minasian L, Kohn EC. New strategies in ovarian cancer treatment. Cancer. 2019;125(Suppl 24):4623–4629. 31967682 10.1002/cncr.32544 PMC7437367

[4] Odunsi K. Immunotherapy in ovarian cancer. Ann Oncol. 2017;28:viii1–viii7. 29232467 10.1093/annonc/mdx444 PMC5834124

[5] García García Y, Marín Alcalá M, Martínez Vila C. Anti-angiogenic therapy for ovarian cancer. EJC Suppl. 2020;15:77–86. 33240446 10.1016/j.ejcsup.2020.02.003 PMC7573465

[6] Guan LY, Lu Y. New developments in molecular targeted therapy of ovarian cancer. Discov Med. 2018;26:219–229. 30695681

[7] Viallard C, Larrivee B. Tumor angiogenesis and vascular normalization: alternative therapeutic targets. Angiogenesis. 2017;20:409–426. 28660302 10.1007/s10456-017-9562-9

[8] Lim D, Do Y, Kwon BS, et al. Angiogenesis and vasculogenic mimicry as therapeutic targets in ovarian cancer. BMB Rep. 2020;53:291–298. 32438972 10.5483/BMBRep.2020.53.6.060 PMC7330806

[9] Ueda M, Terai Y, Kanda K, et al. Tumor angiogenesis and molecular target therapy in ovarian carcinomas. Hum Cell. 2005;18:1–16. 16130895 10.1111/j.1749-0774.2005.tb00052.x

[10] Gómez-Raposo C, Mendiola M, Barriuso J, et al. Angiogenesis and ovarian cancer. Clin Transl Oncol. 2009;11(9):564–571. 19775995 10.1007/s12094-009-0406-y

[11] Grunewald T, Ledermann JA. Targeted therapies for ovarian cancer. Best Pract Res Clin Obstet Gynaecol. 2017;41:139–152. 28111228 10.1016/j.bpobgyn.2016.12.001

[12] Chelariu-Raicu A, Horn A, Jonglertham P. Anti-angiogenesis therapy in ovarian cancer: which patient is it most likely to benefit? Oncology (Williston Park). 2019;33(1):33. 31365748

[13] Pero SC, Daly RJ, Krag DN. Grb7-based molecular therapeutics in cancer. Expert Rev Mol Med. 2003;5(14):1–11. 10.1017/S1462399403006227 14585167

[14] Zeng M, Yang Z, Hu X, et al. Grb7 gene amplification and protein expression by FISH and IHC in ovarian cancer. Int J Clin Exp Pathol. 2015;8:11296–11304. 26617853 PMC4637669

[15] Zheng Y, Pei Y, Yang L, et al. GRB7 promotes proliferation and tumorigenesis of bladder cancer via Phospho-AKT pathway. Int J Biol Sci. 2020;16:3221–3230. 33162827 10.7150/ijbs.49410 PMC7645994

[16] Wang Y, Chan DW, Liu VW. Differential functions of growth factor receptor-bound protein 7 (GRB7) and its variant GRB7v in ovarian carcinogenesis. Clin Cancer Res. 2010;16:2529–2539. 20388850 10.1158/1078-0432.CCR-10-0018

[17] Chen K, Liu MX, Mak CS, et al. Methylation-associated silencing of miR-193a-3p promotes ovarian cancer aggressiveness by targeting GRB7 and MAPK/ERK pathways. Theranostics. 2018;8:423–436. 29290818 10.7150/thno.22377 PMC5743558

[18] Chan DW, Hui WW, Cai PC, et al. Targeting GRB7/ERK/FOXM1 signaling pathway impairs aggressiveness of ovarian cancer cells. PLoS One. 2012;7:e52578. 23285101 10.1371/journal.pone.0052578 PMC3527599

[19] Lertkiatmongkol P, Liao D, Mei H. Endothelial functions of platelet/endothelial cell adhesion molecule-1 (CD31). Curr Opin Hematol. 2016;23(3):253–259. 27055047 10.1097/MOH.0000000000000239 PMC4986701

[20] Schmidt LH, Biesterfeld S, Kümmel A, et al. Tissue microarrays are reliable tools for the clinicopathological characterization of lung cancer tissue. Anticancer Res. 2009;29:201–209. 19331151

[21] Bollag G, Hirth P, Tsai J, et al. Clinical efficacy of a RAF inhibitor needs broad target blockade in BRAF-mutant melanoma. Nature. 2010;467:596–599. 20823850 10.1038/nature09454 PMC2948082

[22] Guo H, Xia B. Collapsin response mediator protein 4 isoforms (CRMP4a and CRMP4b) have opposite effects on cell proliferation, migration, and invasion in gastric cancer. BMC Cancer. 2016;16(1):565. 27475326 10.1186/s12885-016-2593-6 PMC4967517

[23] Xiao J, Chen B, Wang Q. Paeonin extracted from potatoes protects gastric epithelial cells from H(2)O(2)-induced oxidative damage in vitro by PI3K/Akt-mediated Nrf2 signaling pathway. Sci Rep. 2018;8:10865. 30022028 10.1038/s41598-018-28772-5 PMC6052145

[24] Lin J, Cao S, Wang Y, et al. Wang Q and Zheng L. Long non-coding RNA UBE2CP3 enhances HCC cell secretion of VEGFA and promotes angiogenesis by activating ERK1/2/HIF-1α/VEGFA signalling in hepatocellular carcinoma. J Exp Clin Cancer Res. 2018;37(1):113. 29866133 10.1186/s13046-018-0727-1 PMC5987644

[25] Shi M, Chen X, Li H. δ-tocotrienol suppresses the migration and angiogenesis of trophoblasts in preeclampsia and promotes their apoptosis via miR-429/ ZEB1 axis. Bioengineered. 2021;12(1):1861–1873. 34002673 10.1080/21655979.2021.1923238 PMC8806315

[26] Chazotte B. Labeling nuclear DNA using DAPI. Cold Spring Harb Protoc. 2011;2011(1). pdb.prot5556. DOI:10.1101/pdb.prot5556. 21205856

[27] Dai F, Luo F, Zhou R, et al. Calponin 3 is associated with poor prognosis and regulates proliferation and metastasis in osteosarcoma. Aging (Albany NY). 2020;12(14):14037–14049. 32667904 10.18632/aging.103224 PMC7425500

[28] Cheng GY, Jiang Q, Deng AP, et al. CD31 induces inflammatory response by promoting hepatic inflammatory response and cell apoptosis. Eur Rev Med Pharmacol Sci. 2018;22:7543–7550. 30468504 10.26355/eurrev_201811_16296

[29] Nefedova NA, Kharlova OA, Danilova NV. Markers of angiogenesis in tumor growth. Arkh Patol. 2016;78:55–63. 27340718 10.17116/patol201678255-62

[30] Figueiredo CC, Pereira NB, Pereira LX, et al. Double immunofluorescence labeling for CD31 and CD105 as a marker for polyether polyurethane-induced angiogenesis in mice. Histol Histopathol. 2019;34:257–264. 30207375 10.14670/HH-18-038

[31] Xu L, Xu C, Lin X. Interference with lysophosphatidic acid receptor 5 ameliorates oxidized low-density lipoprotein-induced human umbilical vein endothelial cell injury by inactivating NOD-like receptor family, pyrin domain containing 3 inflammasome signaling. Bioengineered. 2021;12(1):8089–8099. 34662522 10.1080/21655979.2021.1983975 PMC8806909

[32] Gordon MS, Mendelson DS, Kato G. Tumor angiogenesis and novel antiangiogenic strategies. Int J Cancer. 2010;126(8):1777–1787. 19904748 10.1002/ijc.25026

[33] Claesson-Welsh L, Welsh M. VEGFA and tumour angiogenesis. J Intern Med. 2013;273(2):114–127. 23216836 10.1111/joim.12019

[34] Zhao Y, Adjei AA. Targeting angiogenesis in cancer therapy: moving beyond vascular endothelial growth factor. Oncologist. 2015;20(6):660–673. 26001391 10.1634/theoncologist.2014-0465 PMC4571783

[35] Lugano R, Ramachandran M, Dimberg A. Ramachandran M and Dimberg A. Tumor angiogenesis: causes, consequences, challenges and opportunities. Cell Mol Life Sci. 2020;77(9):1745–1770. 31690961 10.1007/s00018-019-03351-7 PMC7190605

[36] Melincovici CS, Boşca AB, Şuşman S, et al. Vascular endothelial growth factor (VEGF) - key factor in normal and pathological angiogenesis. Rom J Morphol Embryol. 2018;59:455–467. 30173249

[37] Namiecińska M, Marciniak K, Nowak JZ. Marciniak K and Nowak JZ. [VEGF as an angiogenic, neurotrophic, and neuroprotective factor]. Postepy Hig Med Dosw (Online). 2005;59:573–583. 16407796

[38] Hellström M, Phng LK, Hofmann JJ, et al. Dll4 signalling through Notch1 regulates formation of tip cells during angiogenesis. Nature. 2007;445:776–780. 17259973 10.1038/nature05571

[39] Jakobsson L, Bentley K, Gerhardt H. VEGFRs and Notch: a dynamic collaboration in vascular patterning. Biochem Soc Trans. 2009;37(6):1233–1236. 19909253 10.1042/BST0371233

[40] Buchert M, Burns CJ, Ernst M. Targeting JAK kinase in solid tumors: emerging opportunities and challenges. Oncogene. 2016;35(8):939–951. 25982279 10.1038/onc.2015.150

